# Diversity and Prevalence of Fungi Associated with Freshwater Crayfish in Three Regions of Lithuania: A First Assessment

**DOI:** 10.3390/microorganisms14092054

**Published:** 2026-09-14

**Authors:** Jurgita Švedienė, Gintautas Vaitonis, Vita Raudonienė, Simas Kasputis, Algimantas Paškevičius

**Affiliations:** State Scientific Research Institute Nature Research Centre, Akademijos St. 2, LT-08412 Vilnius, Lithuania; gintautas.vaitonis@gamtc.lt (G.V.); vita.raudoniene@gamtc.lt (V.R.); simas.kasputis@gamtc.lt (S.K.); algimantas.paskevicius@gamtc.lt (A.P.)

**Keywords:** crayfish, fungi, Lithuania, ITS, diversity

## Abstract

Fungi play an important role in freshwater ecosystems, yet their diversity and distribution among crayfish species remain poorly understood. We analyzed the diversity of fungi in four crayfish species (*Astacus astacus*, *Faxonius limosus*, *Pacifastacus leniusculus*, and *Pontastacus leptodactylus*) collected across three regions of Lithuania: northeast, southeast, and west. A total of 27 crayfish were collected, and each crayfish was examined for muscle and hepatopancreas tissues. Of the 27 tested individuals, four crayfish were free of fungi. A total of 77 isolates of fungi were obtained from the hepatopancreas and muscle tissue samples of the examined crayfish using direct isolation. Combining phenotypic characterization with ITS sequencing, 28 fungal genera were identified, with *Cladosporium*, *Fusarium*, and *Penicillium* being the predominant genera. Fungi were more frequent in muscle tissue (81.5%) than in hepatopancreas tissue (66.7%), *p* = 0.219. *A. astacus* and *P. leniusculus* exhibited the highest prevalence of fungi, with 100% of individuals (*n* = 2). Regional diversity of fungi varied across regions, with Shannon diversity values ranging from H′ = 1.748 in the west to H′ = 3.076 in the southeast. These patterns suggest apparent host- and region-associated differences in crayfish fungal communities.

## 1. Introduction

Freshwater is one of the most important and vulnerable resources on Earth. It accounts for only 0.01% of water in the world and about 0.8% of the land surface [1,2]. Freshwater ecosystems provide vital resources for humans and are the sole habitat for an extraordinarily rich, endemic, and sensitive biota [3]. Also, they are exceptionally rich in biodiversity (over 13% of all species; ~30% of all vertebrates) [4]. Unfortunately, biodiversity in freshwater ecosystems declines at a considerably greater rate than in terrestrial ecosystems due to habitat destruction, climate change, and invasive species [4,5]. Crayfish are the largest invertebrates inhabiting freshwater ecosystems. The most widespread crayfish species have broad tolerances to ranges or extremes of temperature, dissolved oxygen and salinity [6,7]. Crayfish play an important role in freshwater ecosystems, including breaking down dead plant material [8,9]. They also serve as bioindicators of water quality and of the health of communities and habitats [6,10,11,12]. Additionally, they stir up and agitate bottom sediments, contributing to the aeration of the bottoms of reservoirs and reducing eutrophication levels [9,13]. Moreover, crayfish are economically important and are cultivated in aquaculture for food and ornamental purposes [14].

Four crayfish species currently inhabit Lithuanian freshwaters: the native *A. astacus*, the translocated *P. leptodactylus*, and the North American invaders *P. leniusculus* and *F. limosus*. *P. leptodactylus* was introduced to Lithuania at the end of the nineteenth century [15]. This introduction was facilitated by the species’ natural distribution, which extends to regions east and southeast of Lithuania. In contrast, *P. leniusculus* was deliberately introduced in 1972 into several closed lakes in the Trakai and Vilnius districts [16]. Much later, *F. limosus* was first recorded in Lithuania in 1994, after which it spread rapidly throughout the country [17]. Over the last decade, approximately 200–*A. astacus*, 130–*P. leptodactylus*, 450–*F. limosus*, and 50–*P. leniusculus* localities have been identified in Lithuania. Most of these localities are lakes, but many crayfish populations are also found in rivers and streams (unpublished data). Also, these same species of crayfish are found in Latvia [18,19], in Poland [20], and in Belarus [21].

Fungi play an essential role in all ecosystem services, including primary production (nutrient creation and enhancement), secondary production (food provisioning and fungi-fauna interactions), and population and community regulation [22]. In aquatic habitats, fungi regulate food web dynamics and biogeochemical cycling and interact with other organisms [23,24]. Freshwater fungi are an important and ubiquitous group of taxa that includes species that spend their entire life cycle, or part of it, in freshwater habitats or that colonize the submerged parts of plants in freshwater environments. These fungi occur across all fungal phyla, but predominantly within the three major groups Ascomycota, Basidiomycota and Chytridiomycota [25]. Most freshwater fungi are associated with organic matter derived from decaying plants and animals [26,27]. They occur in various types of water reservoirs, including freshwater and seawater, running and stagnant waters, and surface and underground waters [28]. Fungi and fungi-like pathogenic species have a particularly significant impact on freshwater ecosystems, causing a global decline in freshwater biodiversity that is far greater than that seen in terrestrial ecosystems [29].

However, limited research has been conducted on the diversity, composition, and dynamics of the fungi and oomycetes that live on and within European freshwater crayfish, especially across native versus invasive hosts, their tissues and compartments, habitats, seasons, and infection states. Only a few papers directly characterize crayfish-associated mycobiomes, and most of those are outside the Baltic basin [30,31,32,33,34,35,36,37,38,39]. Existing studies reveal that crayfish have a broad diversity of fungi and fungus-like organisms that are ecologically significant. Studies on the invasive species *Procambarus clarkii* have shown that they have rich and structured assemblages of fungi in their digestive tracts and on their exoskeletons. These assemblages are shaped by host traits, seasonality, and competitive interactions among fungal taxa [31,32]. Studies on *P. leniusculus* also show that crayfish mycobiomes differ greatly across tissues and are partly structured by environmental communities of fungi. This indicates a dynamic exchange between hosts and their habitats [30]. Studies focused on surveillance provide additional evidence that fungi and oomycete associations vary geographically, temporally, and among host species, even within the context of *Aphanomyces astaci* monitoring [35,36,37,38,39]. These studies underscore the complexity of crayfish-fungi interactions, which extend beyond the presence or absence of the plague pathogen; they point to diverse, structured, and environmentally responsive mycobiomes that remain poorly characterized across most of Europe, including the Baltic region.

This study presents the first detailed analysis of diversity and prevalence of fungi in crayfish across three regions of Lithuania. Through the combination of prevalence data, diversity metrics, bootstrap uncertainty analysis, and organ-level comparisons, our objectives were to quantify fungal diversity patterns across species and geography and identify fungi from muscle and hepatopancreas tissues using ITS sequencing.

## 2. Materials and Methods

### 2.1. Crayfish Sampling

**Figure 1:**
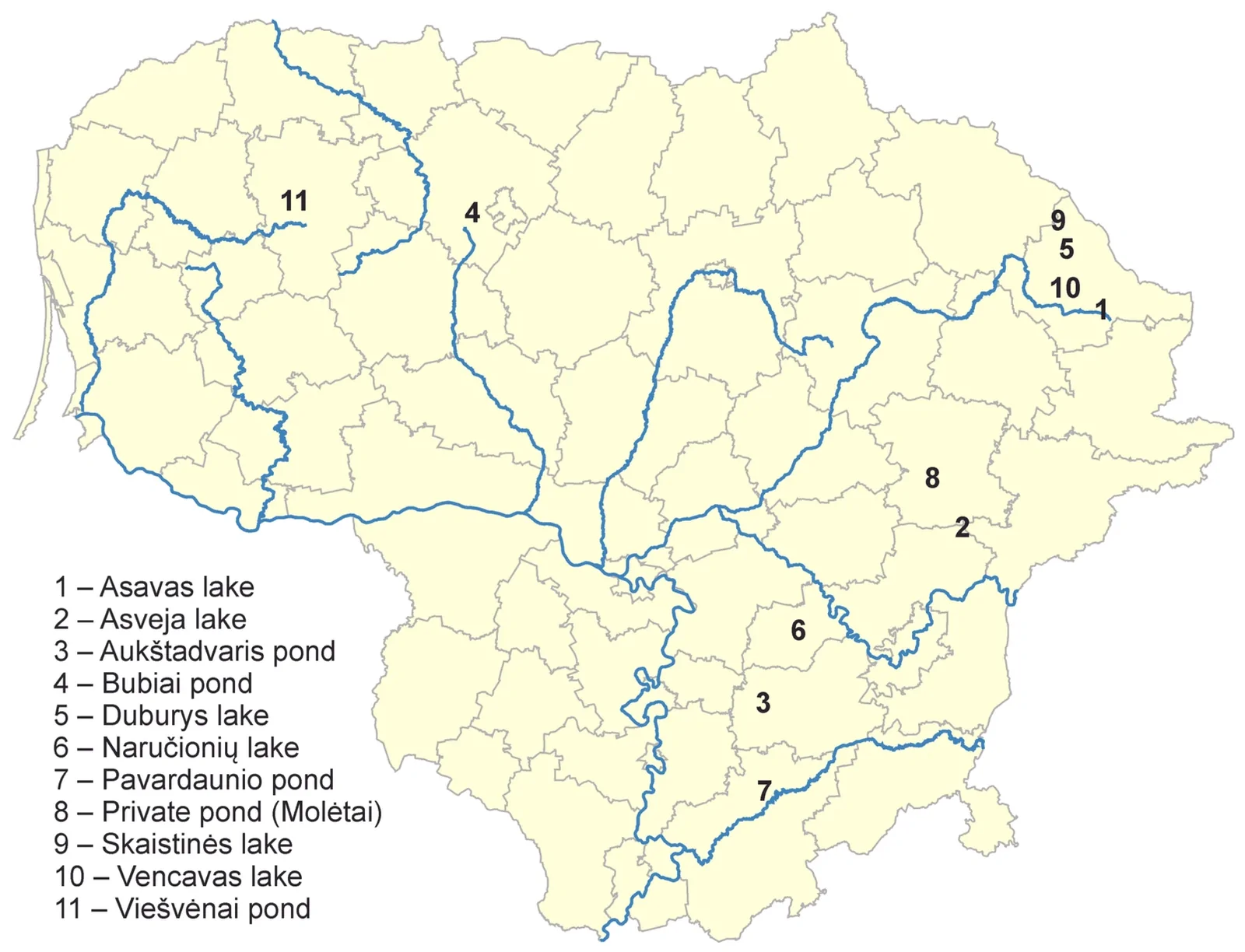
Map of Lithuania with the sampling sites marked by numbers. 1. Asavas lake (55.624112, 26.196097), 2. Asveja lake (55.033655, 25.541768), 3. Aukštadvaris pond (54.577450, 24.553549), 4. Bubiai pond (55.889990, 23.125111), 5. Duburys lake (55.794233, 25.970184), 6. Naručionių lake (54.740346, 24.778440), 7. Pavardaunio pond (54.274974, 24.451479), 8. Private pond (55.153258, 25.338129), 9. Skaistinės lake (55.890652, 25.966922), 10. Vencavas lake (55.713449, 25.976192), 11. Viešvėnai pond (55.932817, 22.311697).

Crayfish were collected in the summer (July and August) of 2025 from 11 locations in three regions of Lithuania: northeast, southeast, and west. A total of 27 healthy crayfish with no injuries (intact appendages) and typical behavior (walking and appendage movements) were collected and examined. The locations where the crayfish were collected are indicated in Figure 1 and Table 1.

### 2.2. Crayfish Organ Sampling

**Figure 2:**
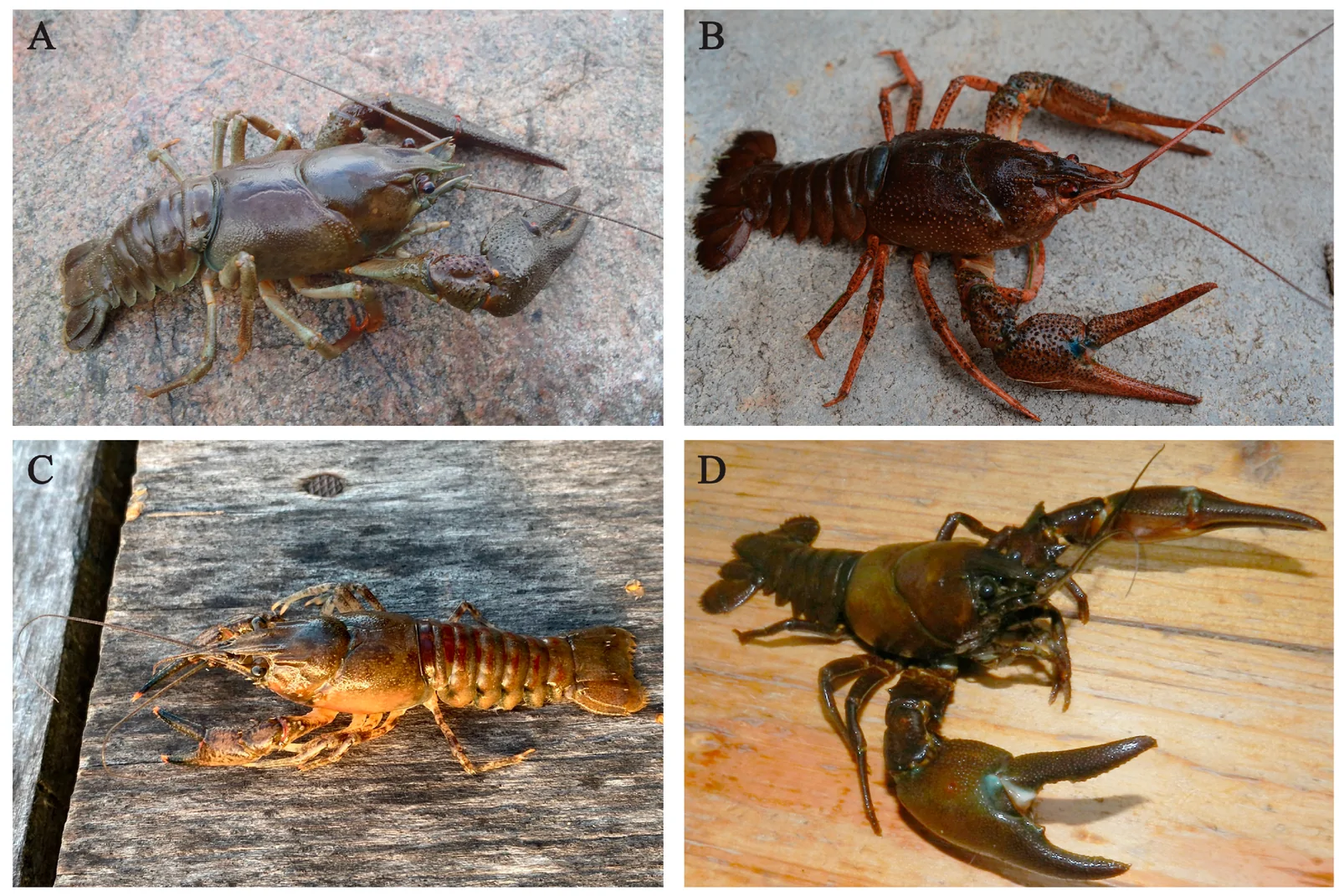
Collected crayfish (**A**) *A. astacus*, (**B**) *P. leptodactylus*, (**C**) *F. limosus*, (**D**) *P. leniusculus*.

In this study, four species of crayfish were included: *A. astacus*, *F. limosus*, *P. leniusculus*, and *P. leptodactylus* (Figure 2). Captured crayfish were placed in sterilized bags individually and kept on ice in a sampling box and immediately transported to the laboratory. The crayfish were kept at 4 °C to slow their activity.

Each animal was killed according to guidelines for the humane killing of crayfish (rapid cut of the nerve cord from the thorax to the end of the abdomen; however, no institutional or national ethical guidelines exist for crayfish) [40]. The surface of the crayfish was sterilized with 70% ethanol for one minute and then washed in sterile distilled water. Each animal was dissected, and the same sampling procedure was used to obtain both the hepatopancreas and the muscles: the complete organ was removed from the body and placed in a sterile Petri dish (27 samples of hepatopancreas tissue and 27 samples of muscle). The sample was washed to minimize external contamination. Non-disposable dissecting equipment was alcohol flame sterilized between each sample.

### 2.3. Isolation of Fungi from Crayfish Tissues

**Figure 3:**
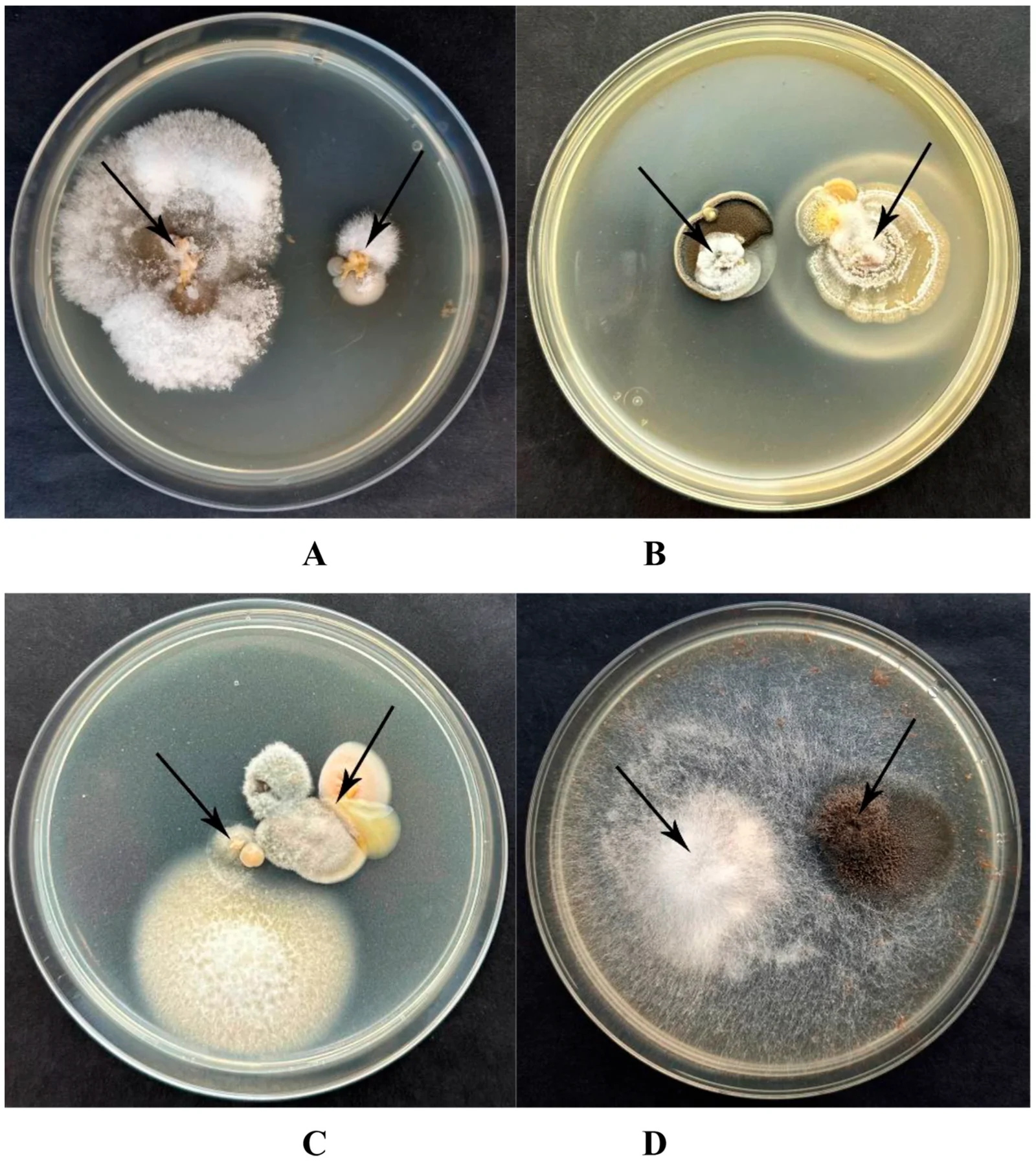
Isolation of fungi from crayfish. (**A**) *F. limosus* muscle tissues on IM agar; (**B**) *P. leniusculus* muscle tissues on IM agar; (**C**) *F. limosus* hepatopancreas tissues on PDA; (**D**) *P. leniusculus* hepatopancreas tissues on PDA. Black arrows indicate tissue samples.

Tissue samples (i.e., the hepatopancreas and muscles) were washed in sterile distilled water (to minimize external contamination) and carefully divided into four parts using a sterile scalpel. The parts were placed on Petri plates containing the medium: (1) IM agar (1.0 g yeast extract, 5.0 g glucose, 20.0 g agar, 1000 mL lake water containing ampicillin (100 µg/mL) and oxolinic acid (100 µg/mL)) [41], and (2) potato dextrose agar (PDA, Liofilchem, Italy) containing streptomycin (100 µg/mL) and ampicillin (100 µg/mL) to inhibit bacterial growth (Figure 3). The plates were incubated at 20 °C for two weeks. Colonies appearing on the agar medium were picked up and isolated onto new PDA plates every two days to obtain pure cultures [42]. The morphology of fungi (branched septate hyphae, pseudohyphae, conidiophores, conidia, poroconidia, arthroconidia and sporangiosphores, etc.) was examined under a LEICA DM750 (Leica, Germany) microscope for identification of isolates following the taxonomic keys for different taxa [43,44,45,46].

### 2.4. Identification of Isolates with ITS rDNA

For the molecular identification, genomic DNA from fungal cultures was extracted using the ZR Fungal/Bacterial DNA MiniPrep kit (Zymo Research, Tustin, CA, USA) according to the manufacturer’s instructions. The isolates were identified by sequencing the Internal Transcribed Spacer region of rDNA, using primers ITS4 and ITS5. PCR amplification was conducted in a 25 µL reaction volume containing the following final concentrations of 2X ReadyMix containing KapaTaq DNA polymerase (1.25 U per 25 µL) (KAPA Biosystems, Wilmington, DE, USA); 0.4 µM of each ITS4 (5′-TCC TCC GCT TAT TGA TAT GC-3′) and ITS5 (5′-GCA AGT AAA AGT CGT AAC AAG G-3′) primers following the protocol described by White et al., 1990 [47]; and 1 µL of template DNA. The target region was amplified by PCR using a Techne TC-5000 thermocycler (Bibby Scientific Ltd., Stone, UK). Amplification was performed with an initial denaturation step at 95 °C for 2 min, followed by 35 cycles of denaturation at 95 °C for 30 s, annealing at 55 °C for 30 s, extension at 72 °C for 1 min, and a final extension at 72 °C for 10 min. The PCR products were purified using Exonuclease I and Shrimp Alkaline Phosphatase (Thermo Scientific, Waltham, USA), according to the protocol described by Stepień et al. (2019) [48]. Base-Clear B.V. (Leiden, The Netherlands) performed sequencing of the purified PCR products. PCR products were sequenced in both directions. The obtained sequences were assembled and edited using the DNA LaserGene SeqMan Pro program (DNASTAR, Madison, WI, USA). For species identification, sequences (555 bp from hepatopancreas, 574 bp from muscles) were compared with publicly available sequences in the National Center for Biotechnology Information (NCBI; https://blast.ncbi.nlm.nih.gov/Blast.cgi) accessed on 20 February 2025 database with BLAST 2.17.0 algorithm. Two sequences were considered to belong to the same species if they showed at least 99% similarity. The newly generated sequences were deposited in GenBank (https://www.ncbi.nlm.nih.gov), and their accession numbers are listed in Table S1.

### 2.5. Phylogenetic Analysis

Sequence data obtained in the laboratory were compared with sequence data acquired from GenBank database (https://www.ncbi.nlm.nih.gov) (Table S1). Evolutionary analyses were conducted using MEGA 12 software. The evolutionary history was inferred through maximum likelihood employing the Tamura-Nei model [49]. The trees with 1000 bootstrap replicates illustrated the similarity of the isolates. *Saccharomyces cerevisiae* Meyen ex E.C. Hansen CBS 1171 was used as the outgroup. Positions with gaps were excluded from analysis.

### 2.6. Data Analysis

Prevalence of fungi in muscle and hepatopancreas tissue was compared using paired data, as both organs were sampled from each of the 27 individuals. Discordant pairs were evaluated with McNemar’s exact test. Wilson 95% confidence intervals were calculated for each species, region and organ-level prevalence.

Shannon diversity (H′) was calculated for each crayfish species and geographic region using full lists of the community of fungi aggregated at the individual level. For each group, taxa from all individuals were pooled to generate community-level diversity. To estimate uncertainty in diversity metrics, we performed 5000 bootstrap resamples for each species and region. Individuals were drawn with replacement, and Shannon diversity was recalculated. From these distributions, we extracted the bootstrap mean and 95% confidence interval (CI).

All calculations were performed using PAST version 5.0.2 (Natural History Museum, University of Oslo, Oslo, Norway).

## 3. Results

Fungi were isolated from muscle and hepatopancreas tissues of crayfish collected from three geographic regions of Lithuania. Of the 27 crayfish examined, only four were free of fungi. Fungi were isolated from muscle tissue in 22 crayfish (81.5%, 95% CI: 63.3–91.8%) and from hepatopancreatic tissue in 18 (66.7%, 95% CI: 47.8–81.4%) of the 27 crayfish examined. Among the six individuals with discordant results, fungi were detected only in muscle tissue in five individuals, whereas in one individual, fungi were detected only in the hepatopancreas. Although fungi were recovered from muscle more often, the difference was not statistically significant (McNemar’s exact test, *p* = 0.219), reflecting the small number of discordant observations.

In total, 77 isolates of fungi were obtained from the hepatopancreas and muscle tissue of the crayfish examined. During the investigation, 64 isolates of fungi were identified; 13 strains remained unidentified because the obtained sequences either showed insufficient similarity to reference sequences in public databases or generated ambiguous alignment results that prevented confident taxonomic assignment. In total, combining phenotypic characterization with ITS sequencing, 28 genera were identified. The identified isolates represented 28 genera, of which 25 belonged to the phylum Ascomycota, with 18 named species identified within this phylum (Table 2).

Fungi from *Ascomycota* belonged to *Eurotiomycetes*, *Dothideomycetes*, *Sordariomycetes*, and *Saccharomycetes* classes. *Dothideomycetes* was the dominant class, with *Pleosporales* order with 12 genera including *Alternaria, Comoclathris, Didymella, Didymocyrtis, Epicoccum, Neocucurbitaria, Neodidymelliopsis*, *Neosetophoma*, *Paracamarosporium*, *Paraphaeosphaeria*, *Phoma*, *Septoriella*. Basidiomycota was represented by 2 strains of *Coprinellus disseminatus* (PZ469777, PZ469778), *Schizophyllum commune* (PZ469779) and *Coriolopsis* sp. strains.

**Figure 4:**
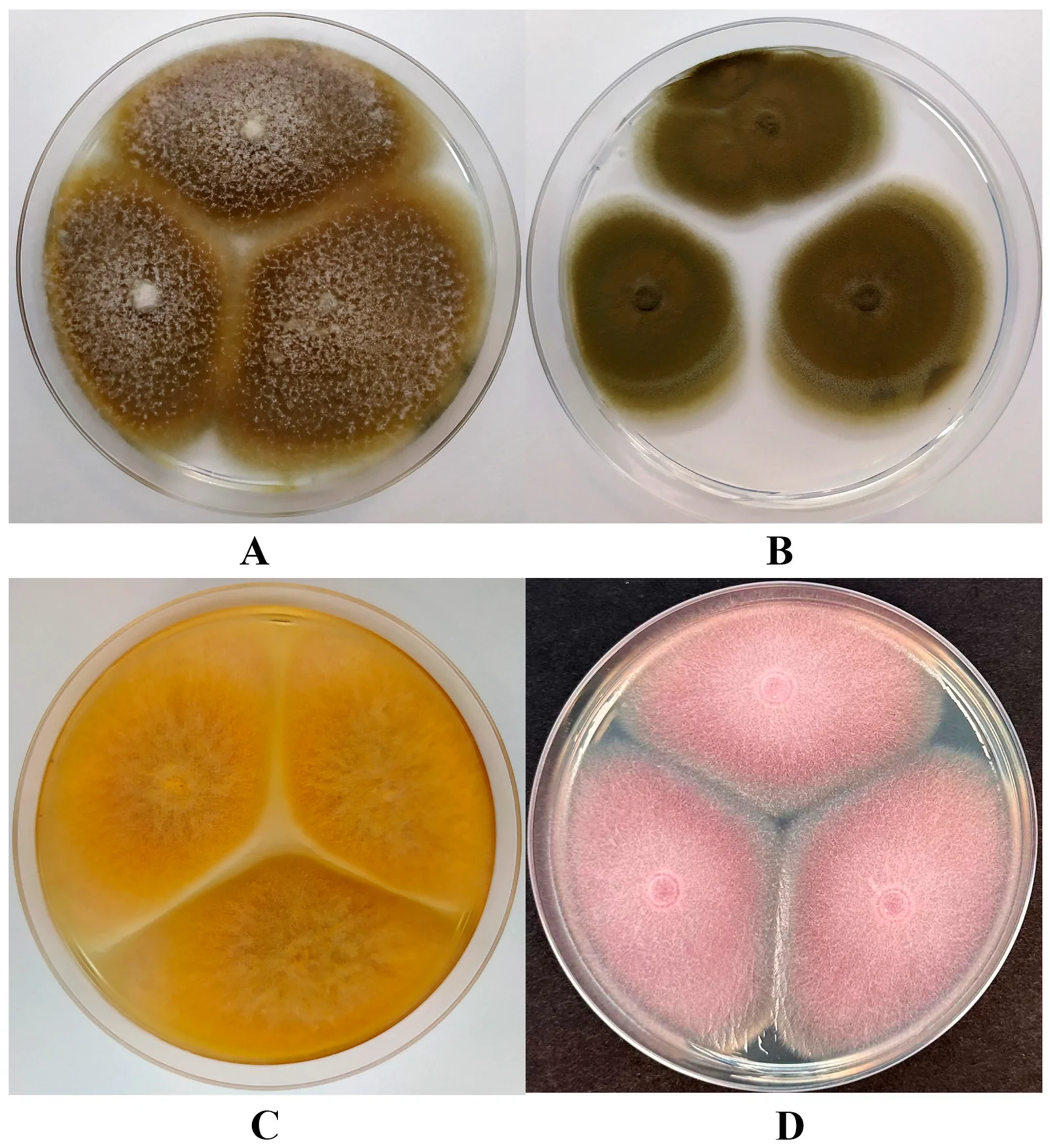
Representative morphological characteristics of fungal isolates (**A**–**D**). (**A**) *Alternaria* sp., (**B**) *C. cladosporioides*, (**C**) *Epicoccum tritici*, (**D**) *F. foetens*. Colony morphology on PDA medium after 7 days of incubation.

**Figure 5:**
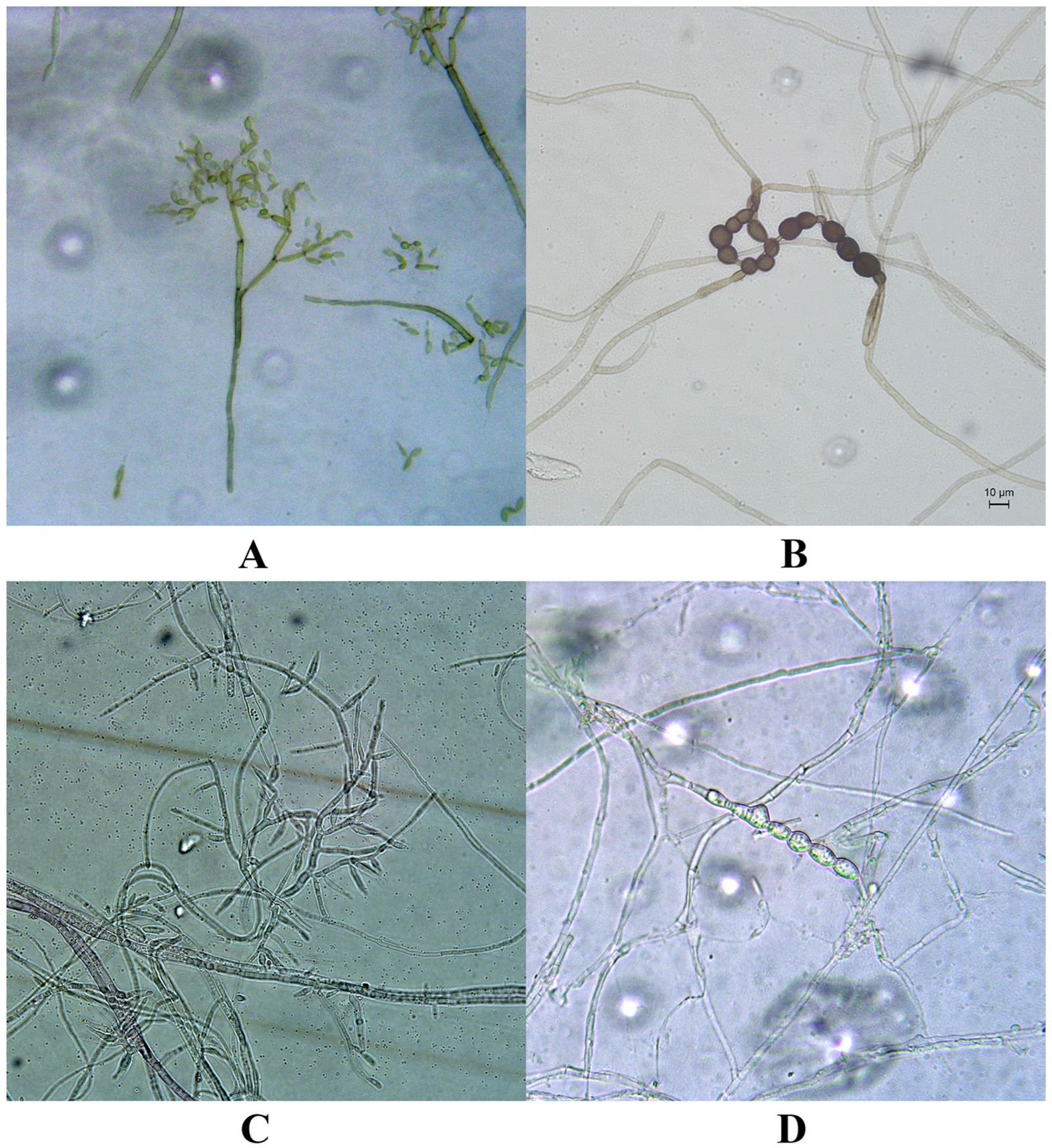
Representative microscopic characteristics of fungal isolates showing hyphae, conidia, and other diagnostic structures. (**A**–**D**). (**A**) *Cladosporium* sp., (**B**) *Didymella* sp., (**C**) *F. foetens*, (**D**) *Fusarium* sp. Scale bar = 10 μm.

Morphological examination supported the molecular identification of fungal isolates. Colonies displayed species-specific pigmentation, texture, and growth patterns, while microscopic observations revealed diagnostic hyphal and conidial characteristics. Representative colony and microscopic features are presented in Table 3 and Figure 4 and Figure 5.

A total of 64 fungal ITS sequences were analyzed and deposited in GenBank under PX-series accession numbers and some PZ-series. The number of identified isolates was obtained from *A. astacus* (25), followed by *F. limosus* (20), *P. leniusculus* (12), and *P. leptodactylus* (7) (Supplementary Table S1). *Cladosporium* was the most frequently identified genus in all four crayfish species. Other fungal taxa showed a more distinct distribution among crayfish species. *Fusarium* was most frequently found in *A. astacus*, including three isolates of *F. foetens*, which were found only in *A. astacus*. Moreover, all *Didymella* isolates were also isolated from *A. astacus*. In *F. limosus*, *Penicillium* was also commonly isolated together with *Cladosporium*. Both *F. sporotrichioides* isolates, *Microdochium bolleyi*, *Didymocyrtis pini*, and *Tolypocladium* sp. were obtained from *P. leniusculus*. Fungal isolates were more frequently obtained from muscle tissue in *A. astacus* and *F. limosus*, while in *P. leniusculus* most isolates were recovered from hepatopancreas tissue.

**Figure 6:**
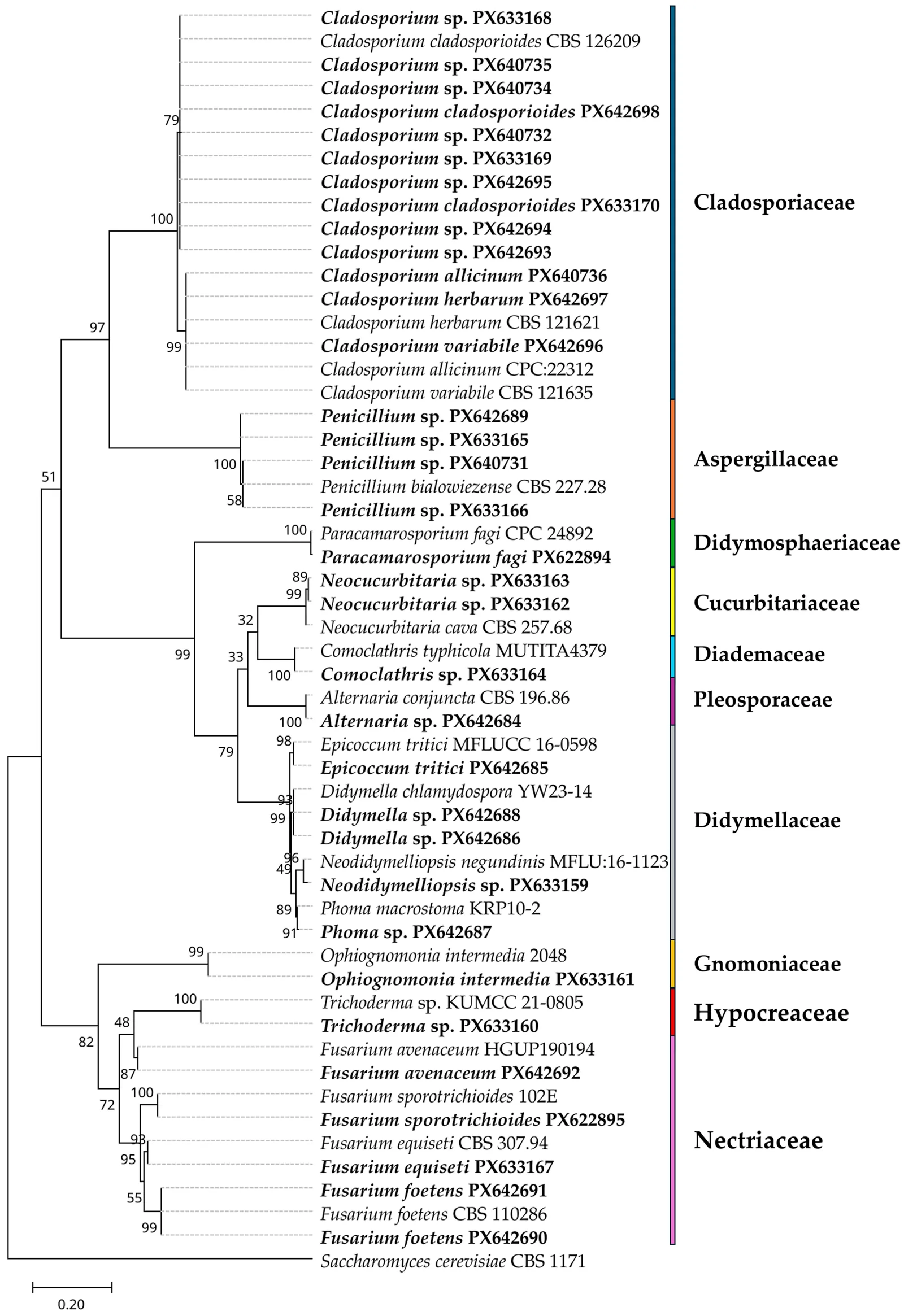
Phylogenetic relationships among fungi isolated from crayfish muscle tissues. Newly generated sequences in bold.

**Figure 7:**
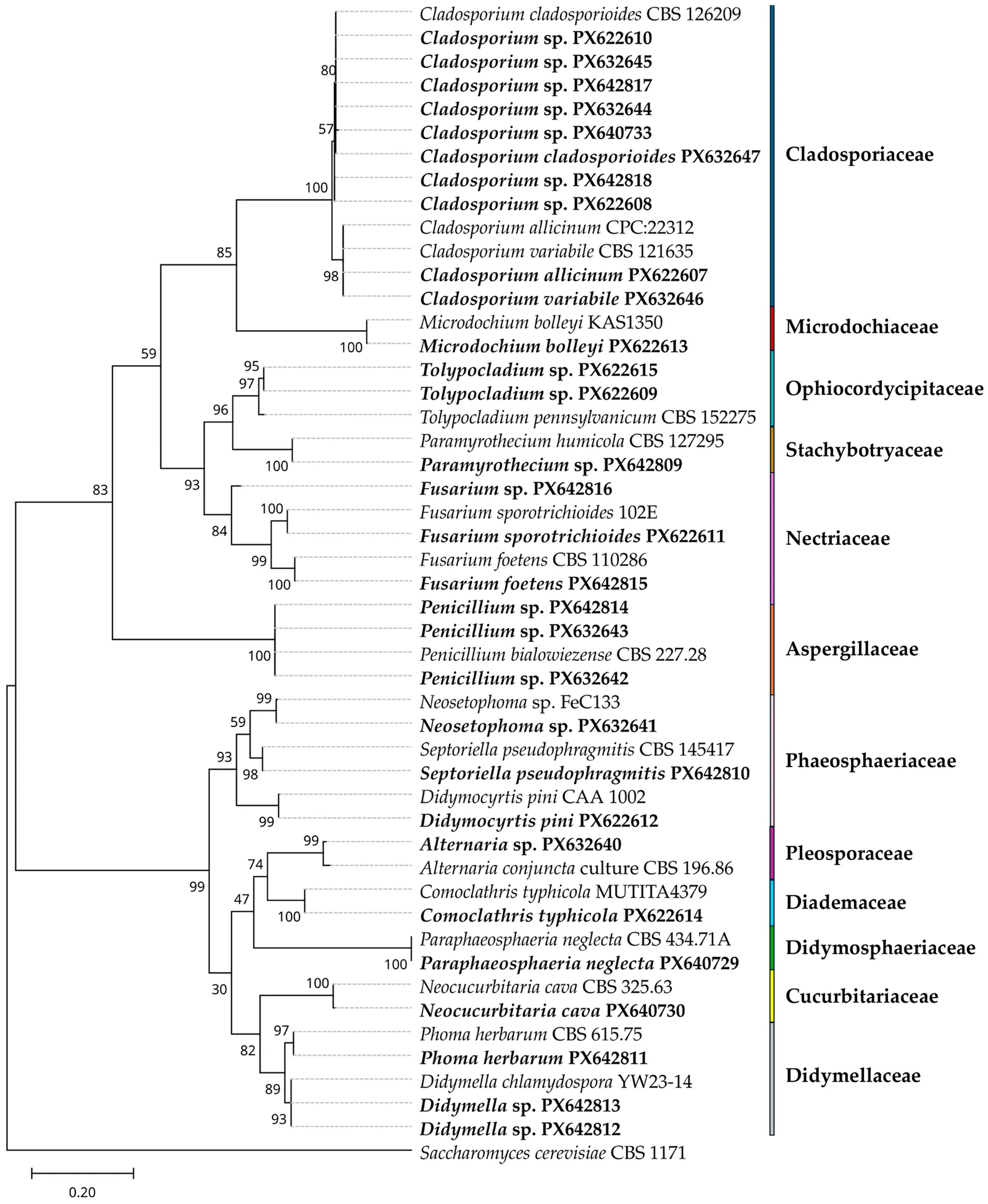
Phylogenetic relationships among fungi isolated from crayfish hepatopancreas tissues. Newly generated sequences in bold.

The phylogenetic tree was constructed to compare the genetic distance between the *Ascomycota* fungi that were isolated in this study. Two phylogenetic trees were constructed for fungi isolated from muscle and from hepatopancreas tissues (Figure 6 and Figure 7). Bootstrap values are generally high (>90%) at major branches, indicating reliable phylogenetic relationships (Figure 6 and Figure 7). The tree showed broad taxonomic sampling across plant-pathogenic and saprophytic fungi. The *Cladosporium* clade formed a large, well-supported monophyletic group (100% bootstrap) that contained multiple species, including *C. cladosporioides*, *C. herbarum*, *C. allicinum*, and *C. variabile*. *C. cladosporioides* was isolated from both muscle and hepatopancreas tissues of the tested crayfish. *Fusarium* formed a large, well-defined group containing *F. avenaceum*, *F. sporotrichioides*, *F. equiseti*, and *F. foetens* species. The genus showed clear species-level separations with high bootstrap support (93–100%). *F. foetens* was obtained from *A. astacus* muscles and hepatopancreas tissues.

Fungi were widespread among all examined crayfish species, though patterns were evident at both the species and regional levels. At the species level, *A. astacus* and *P. leniusculus* exhibited the highest prevalence of fungi, with 100% of individuals detected (Table 4). The estimate for *P. leniusculus* was based on only two individuals and was therefore accompanied by a wide confidence interval. The other two species, *P. leptodactylus* and *F. limosus*, exhibited a lower prevalence of fungi.

None of the comparisons of fungal prevalence among crayfish species reached statistical significance at the 5% level, although several showed trends reflecting the patterns observed in raw prevalence (*F. limosus* and *A. astacus*, *p* = 0.206; *F. limosus* and *P. leptodactylus*, *p* = 0.585; *P. leptodactylus* and *A. astacus*, *p* = 0.438). These differences likely stem from a combination of biological variation and small sample sizes.

Regional fungi prevalence patterns largely mirrored the results at the crayfish species level (Table 5). The prevalence of fungi was high in both the northeast and the southeast regions, indicating high fungal occurrence across much of Lithuania. In contrast, the west exhibited a lower prevalence of 66.7%, reflecting lower fungal distribution in this region. This pattern was consistent with diversity analyses, in which the west also showed the lowest fungal richness. However, due to the small sample sizes, none of the regional contrasts in prevalence are statistically significant, despite consistent descriptive patterns (lower prevalence in the west) across analyses. This lack of statistical significance reflects a limited sample size within regions rather than an absence of biological differences.

All four crayfish species exhibited diverse fungal communities based on the total fungal community per individual (multiple fungi permitted), with *A. astacus* exhibiting the greatest diversity (Table 6).

*P. leniusculus* had relatively high diversity but wide confidence intervals (CIs) due to a small sample size, while *P. leptodactylus* had the widest CI, indicating less certainty in diversity. Even after accounting for bootstrap uncertainty, substantially higher fungal community diversity was found in southeastern Lithuania (Table 7).

Consistent with the prevalence of fungi summaries, the west showed markedly lower fungi diversity and the narrowest distribution. The southeast region exhibited the highest fungi diversity and the widest bootstrap distribution, which contained a richer and more variable fungi community. The northeast displayed intermediate values, while the west exhibited the lowest fungi diversity and the narrowest distribution. These patterns align with the prevalence analyses and suggest regional differences in fungal diversity.

## 4. Discussion

This study provides the first assessment to date of fungal diversity in Lithuanian crayfish using molecular and morphological identification of isolated strains. Freshwater crayfish are among the largest invertebrates, and where they occur, they often form a major part of benthic biomass [7]. Crayfish play an important role in regulating ecosystem activity, contributing to the aeration of lake bottoms and reducing eutrophication levels. Also, crayfish can serve as hosts to many parasites and symbionts, including viruses, bacteria, fungi, and helminths [50]. Microscopic fungi are the fundamental component of any ecosystem. In freshwater, fungi can reach relative abundances of more than 50% of all eukaryotic sequences [26,51]. Aquatic fungi are broadly defined as fungi that depend on aquatic habitats for all or part of their life cycle. They occur across all fungal phyla but are represented predominantly by members of the Ascomycota, Basidiomycota, and Chytridiomycota [25,52,53]. Beyond their taxonomic diversity, aquatic fungi play important ecological roles in freshwater ecosystems, where they regulate food-web dynamics and biogeochemical cycling and contribute to habitat engineering and stress responses [26,54]. Although research on the distribution and biodiversity of freshwater fungi has increased in recent years, our understanding of their diversity, ecology, and biogeographical patterns remains limited.

Crayfish, like other organisms, can become a habitat for microscopic fungi. In this study, we tested 27 healthy crayfish. The term “healthy” refers to crayfish that showed no visible signs of disease, injury, or abnormal behavior during sampling. Of the 27 tested crayfish, 77 fungal isolates were obtained from hepatopancreas and muscle tissue; only four crayfish were free of fungi. The prevalence of fungi in the muscle tissue of crayfish was 81.5% (95% CI: 63.3–91.8%) and in the hepatopancreas tissue was 66.7% (95% CI: 47.8–81.4%). Fungi and fungus-like organisms were reported in the literature from muscle and other tissues [55]. Fungi may enter the body of crayfish through wounds or to cause secondary infections in wounded or immunocompromised crustaceans [56]. Ascomycota (93.5%) and Basidiomycota (6.5%) fungi were identified from the muscle and hepatopancreas tissues of crayfish using ITS sequencing and phenotypic characterization. According to the literature, Ascomycota and Basidiomycota have been frequently isolated from various crustaceans, including *Eriocheir sinensis* [57], *Procambarus clarkii* [31], *P. leniusculus* [30], and *Portunus sanguinolentus* [58]. Species of fungi belonging to 28 genera were identified from the muscles and hepatopancreas tissues of the studied crayfish species. Among them, *Alternaria*, *Aspergillus*, *Fusarium*, *Penicillium*, *Didymocyrtis*, *Comoclathris*, and *Didymella* have been previously isolated from freshwater habitats [53,59]. *C. cladosporioides* was the dominant species, which was isolated from both muscle and hepatopancreas tissue samples of the tested crayfish. In crayfish, *Cladosporium* species typically occur as saprobic fungi or a common environmental contaminant on the animal’s outer shell [31]. *Fusarium* species (*F. avenaceum* and *F. equiseti*) were only present in the muscle tissue of the tested crayfish. *Fusarium* spp. are common fungi with worldwide distribution, found in soil and freshwater, and are well-known plant pathogens [60]. Also, *Fusarium* spp. have been reported to cause melanization of the shell and gills in crustaceans [61]. *F. avenaceum* isolated from *A. astacus* could cause burn spot disease of crayfish [42]. Additionally, *Fusarium* spp. were described as saprophytes or superficial contaminants on *Austropotamobius pallipes* [62]. The recovery of fungal taxa that include species reported as opportunistic pathogens does not necessarily indicate active infection. These fungi may occur as environmental colonizers, commensals, or asymptomatic infections and may only become pathogenic under specific host or environmental conditions. Therefore, the ecological role and pathogenic significance of the isolated fungi remain uncertain and cannot be inferred from the present study. The pathogenicity of the isolated fungi has not been investigated. However, it is possible that some fungi found are present on crayfish but do not present a risk to that species in that locality [63]. Further investigations, including histopathological analyses and experimental infection studies, are needed to determine whether these fungi represent commensal associates, transient colonizers, or potential pathogens of crayfish.

The native species *A. astacus* had the highest diversity of fungi and a consistently high prevalence, which suggests either broad susceptibility or continuous exposure to environmental fungal reservoirs. In contrast, invasive species, such as *F. limosus*, exhibited region-specific patterns of fungal patterns, notably a reduced presence in the west. Regional variation in prevalence and diversity suggests a strong environmental influence. The southeast region, which had the richest communities of fungi, may have environmental conditions that support a wider variety of fungi, such as differences in temperature, organic matter availability, or hydrological connectivity. The lower diversity of fungi observed in the west may be due to ecological characteristics such as lower productivity, cooler waters, or smaller fungal source populations.

Crayfish are considered keystone species in freshwater ecosystems, and factors affecting their survival can influence food web structure and ecosystem stability. The present study suggests that microscopic fungi may represent an overlooked component of crayfish-associated biota, with potential implications for host health and population dynamics. Nevertheless, the findings should be interpreted cautiously because the study is preliminary, the sample size is limited and uneven, and culture-based methods capture only a subset of fungal diversity. Despite these limitations, cultivation-based techniques provide valuable living isolates for further ecological and pathogenicity studies.

## 5. Conclusions

The study provides the first characterization of the fungal assemblages associated with freshwater crayfish in Lithuania. Crayfish of all four species presented rich and taxonomically diverse fungal communities dominated by Ascomycota—most frequently *Cladosporium*, *Fusarium* and *Penicillium*—with a minor Basidiomycota component. Fungi were recovered more often from muscle than from hepatopancreas tissue. Although *A. astacus* and the southeast region were associated with relatively high diversity values, these patterns were not statistically significant and may have been influenced by the limited sample size. Future research combining culture-dependent and culture-independent approaches, such as ITS metabarcoding and high-throughput sequencing, will be essential for achieving a more comprehensive understanding of crayfish-associated fungal communities and their ecological significance. Such knowledge may ultimately contribute to improved monitoring and conservation of freshwater ecosystems.

## Figures and Tables

**Table 1:** Description of the sampled locations in Lithuania.

Sampling Locations	Species	Area (ha)	Length of the Coastline (km)	Maximum Depth (m)	Average Depth (m)
Asavas lake	*F. limosus* (*n* = 1)	205.5	10.5	6.6	4.2
Asveja lake	*F. limosus* (*n* = 1), *P. leniusculus* (*n* = 1)	1023.4	73.7	50.2	14.7
Aukštadvaris pond	*P. leniusculus* (*n* = 1)	293.5	24.9	40.0	6.6
Bubiai pond	*F. limosus* (*n* = 3)	415.4	47.1	21.0	5.1
Duburys lake	*F. limosus* (*n* = 3)	101.1	11.7	19.4	5.2
Naručionių lake	*F. limosus* (*n* = 1)	16.4	2.2	4.4	2.2
Pavardaunio pond	*P. leptodactylus* (*n* = 3)	11.3	2.4	4.0	2.4
Private pond	*A. astacus* (*n* = 3)	0.1	0.2	3.0 *	1.5 *
Skaistinės lake	*A. astacus* (*n* = 3)	26.3	2.8	6.5	2.4
Vencavas lake	*P. leptodactylus* (*n* = 4)	227.1	7.4	48.4	13.9
Viešvėnai pond	*A. astacus* (*n* = 3)	20.2	4.2	6.0	2.2

* According to the pond owner.

**Table 2:** Isolated fungi across four crayfish species collected in Lithuanian freshwater.

Crayfish Species	N	Fungi
*Astacus astacus*	9	*Alternaria* sp. (1), *Fusarium avenaceum* (1), *Fusarium foetens* (4), *Fusarium* sp. (1), *Cladosporium variabile* (1), *Cladosporium cladosporioides* (1), *Cladosporium herbarum* (1), *Didymella* sp. (3), *Didymella chlamydospora* (1), *Paramyrothecium* sp. (1), *Phoma herbarum* (1), *Phoma* sp. (1), *Penicillium* sp. (3), *Cladosporium* sp. (3), *Epicoccum tritici* (1), *Septoriella pseudophragmitis* (1).
*Faxonius limosus*	9	*Cladosporium* sp. (3), *Cladosporium cladosporioides* (2), *Cladosporium variabile* (1), *Coprinellus disseminatus* (1), *Ophiognomonia intermedia* (1), *Fusarium equiseti* (1), *Geotrichum* sp. (1), *Neodidymelliopsis* sp. (1), *Neocucurbitaria* sp. (1), *Neosetophoma* sp. (1), *Penicillium* sp. (3), *Trichoderma* sp. (1), *Comoclathris* sp. (1), *Alternaria* sp. (1), *Schizophyllum commune* (1).
*Pontastacus leptodactylus*	7	*Aspergillus* sp. (1), *Coprinellus disseminatus* (1), *Cladosporium* sp. (1), *Cladosporium allicinum* (1), *Scopulariopsis brevicaulis* (1), *Emericellopsis* sp. (1), *Paraphaeosphaeria neglecta* (1).
*Pacifastacus leniusculus*	2	*Paracamarosporium fagi* (1), *Coriolopsis* sp. (2), *Cladosporium allicinum* (1), *Cladosporium* sp. (2), *Tolypocladium* sp. (1), *Fusarium sporotrichioides* (2), *Didymocyrtis pini* (1), *Microdochium bolleyi* (1), *Comoclathris* sp. (1).

**Table 3:** Morphological characteristics of fungal species isolated from crayfish tissues.

Species	Representative Strains	Colony Morphology (PDA)	Hyphal Characteristics	Conidia/Spores
*Alternaria* sp.	46 G-288, 78 G-289	Dark green, cottony colony.	Septate, pigmented.	Dark, with septa, ellipsoid.
*Cladosporium allicinum*	33 G-230, 67 G-229	Dark green, mixture of dark and light green, velvety colony.	Septate, pigmented.	Dark, different shapes.
*Cladosporium cladosporioides*	20 G-227, 44 G-226	Dark green, velvety colony.	Septate, pigmented.	Dark, different shapes.
*Fusarium foetens*	1 G-255, 3 G-254, 4 G-523	Pink, cottony.	Hyaline, septate.	Macroconidia and microconidia.
*Didymella chlamydospora*	6 G-263, 7 G-264	Greenish-brown.	Dark, septate.	Ovoid
*Epicoccum tritici*	39 G-287	Pale orange, cottony.	Septate.	Dark, globose.
*Trichoderma* sp.	56 G-284	Light and dark green, cottony colony.	Hyaline, septate.	Subglobose.
*Penicillium* sp.	50 G-258, 80 G-256	Green, powdery colony.	Hyaline, septate.	Conidia in chains, globose.

**Table 4:** Prevalence of fungi by crayfish species.

Species	Total	Isolated from	Prevalence	95% CI
*Astacus astacus*	9	9	1.00	0.701–1.000
*Faxonius limosus*	9	6	0.67	0.354–0.879
*Pacifastacus leniusculus*	2	2	1.00	0.342–1.000
*Pontastacus leptodactylus*	7	6	0.86	0.487–0.974

**Table 5:** Prevalence of fungi in crayfish by region.

Region	Total	Isolated from	Prevalence	95% CI
Northeast	11	10	0.91	0.623–0.984
Southeast	10	9	0.90	0.596–0.982
West	6	4	0.67	0.300–0.903

**Table 6:** Bootstrapped Shannon diversity (H′) of communities of fungi across crayfish species, based on 5000 resamples with corresponding 95% confidence intervals.

Species	Observed H′	Bootstrap Mean	95% CI (2.5–97.5%)
*Astacus astacus*	2.648	2.260	1.721–2.600
*Faxonius limosus*	2.359	2.059	1.560–2.337
*Pacifastacus leniusculus*	2.272	1.998	1.609–2.272
*Pontastacus leptodactylus*	2.205	1.818	1.074–2.220

**Table 7:** Bootstrapped Shannon diversity (H′) of fungal communities across regions, based on 5000 resamples with corresponding 95% confidence intervals.

Region	Observed H′	Bootstrap Mean	95% CI (2.5–97.5%)
Northeast	2.464	2.179	1.788–2.449
Southeast	3.076	2.688	2.224–2.997
West	1.748	1.332	0.693–1.748

## Data Availability

Data available from the corresponding author, J.Š., on request. The newly generated sequences were deposited in GenBank.

## Supplementary Materials

The following supporting information can be downloaded at: https://www.mdpi.com/article/10.3390/microorganisms14092054/s1, Table S1: Species name, strain numbers, and GenBank accession numbers included in phylogenetic analyses. Entries in bold represent newly generated materials.
