# Supplementary material for: Diversity and Prevalence of Fungi Associated with Freshwater Crayfish in Three Regions of Lithuania: A First Assessment
Source: Microorganisms. 2026 Sep 14;14(9):2054. doi: 10.3390/microorganisms14092054 (PMC13609898; doi:10.3390/microorganisms14092054)
Supplement: Supplementary file 1 [file microorganisms-14-02054-s001.zip › microorganisms-4542678-supplementary.pdf]

Table S1. Species name, strain numbers, and GenBank accession numbers included in phylogenetic analyses. Entries in bold represent newly generated materials.

Species	Strain identification number	Crayfish species and the tissue	The country in which the strain was identified	GenBank accession numbers, ITS gene
Alternaria conjuncta	CBS 196.86		The Netherlands	MH861940
***Alternaria* sp.**	**46 G-288**	***Astacus astacus*, muscle**	**Lithuania**	**PX642684**
***Alternaria* sp.**	**78 G-289**	***Faxonius limosus*, liver**	**Lithuania**	**PX632640**
Cladosporium allicinum	CPC:22312		The Netherlands	MF472901
Cladosporium allicinum	**67 G-229**	***Pacifastacus leniusculus*, liver**	**Lithuania**	**PX622607**
Cladosporium allicinum	**33 G-230**	***Pontastacus leptodactylus*, muscle**	**Lithuania**	**PX640736**
Cladosporium cladosporioides	CBS 126209		The Netherlands	MH863979
Cladosporium cladosporioides	**59 G-225**	***Faxonius limosus*, liver**	**Lithuania**	**PX632647**
Cladosporium cladosporioides	**44 G-226**	***Astacus astacus*, muscle**	**Lithuania**	**PX642698**
Cladosporium cladosporioides	**20 G-227**	***Faxonius limosus*, muscle**	**Lithuania**	**PX633170**
***Cladosporium* sp.**	**57 G-233**	***Faxonius limosus*, liver**	**Lithuania**	**PX632645**
***Cladosporium* sp.**	**51 G-234**	***Astacus astacus*, liver**	**Lithuania**	**PX642818**
***Cladosporium* sp.**	**28 G-235**	***Faxonius limosus*, liver**	**Lithuania**	**PX632644**
***Cladosporium* sp.**	**48 G-236**	***Astacus astacus*, liver**	**Lithuania**	**PX642817**
***Cladosporium* sp.**	**49 G-237**	***Astacus astacus*, muscle**	**Lithuania**	**PX642695**
***Cladosporium* sp.**	**41 G-238**	***Astacus astacus*, muscle**	**Lithuania**	**PX642694**

***Cladosporium* sp.**	**37 G-239**	***Pacifastacus leptodactylus*, muscle**	**Lithuania**	**PX640735**
***Cladosporium* sp.**	**71 G-240**	***Pacifastacus leniusculus,* liver**	**Lithuania**	**PX622610**
***Cladosporium* sp.**	**36 G-241**	***Pontastacus leptodactylus*, muscle**	**Lithuania**	**PX640734**
***Cladosporium* sp.**	**14 G-242**	***Faxonius limosus*, muscle**	**Lithuania**	**PX633169**
***Cladosporium* sp.**	**12 G-243**	***Astacus astacus*, muscle**	**Lithuania**	**PX642693**
***Cladosporium* sp.**	**83 G-244**	***Pontastacus leptodactylus,* liver**	**Lithuania**	**PX640733**
***Cladosporium* sp.**	**68 G-245**	***Pacifastacus leniusculus,* liver**	**Lithuania**	**PX622608**
***Cladosporium* sp.**	**34 G-246**	***Pontastacus leptodactylus*, muscle**	**Lithuania**	**PX640732**
***Cladosporium* sp.**	**23 G-247**	***Faxonius limosus,* muscle**	**Lithuania**	**PX633168**
Cladosporium herbarum	CBS 121621		The Netherlands	OK445560
Cladosporium herbarum	**47 G-228**	***Astacus astacus*, muscle**	**Lithuania**	**PX642697**
Cladosporium variabile	CBS 121635		The Netherlands	MH863131
Cladosporium variabile	**21 G-231**	***Faxonius limosus*, liver**	**Lithuania**	**PX632646**
Cladosporium variabile	**5 G-232**	***Astacus astacus*, muscle**	**Lithuania**	**PX642696**
Comoclathris typhicola	MUT<ITA>:4379		Italy	KC339229.1
Comoclathris typhicola	**75 G-269**	***Pacifastacus leniusculus*, liver**	**Lithuania**	**PX622614**
***Comoclathris* sp.**	**77 G-271**	***Faxonius limosus*, muscle**	**Lithuania**	**PX633164**
Didymella chlamydospora	YW23-14		South Korea	MK836111
Didymella chlamydospora	**7 G-264**	***Astacus astacus*, liver**	**Lithuania**	**PX642813**
***Didymella* sp.**	**6 G-263**	***Astacus astacus*, muscle**	**Lithuania**	**PX642688**

***Didymella* sp.**	**11 G-266**	***Astacus astacus*, muscle**	**Lithuania**	**PX642686**
***Didymella* sp.**	**9 G-267**	***Astacus astacus*, liver**	**Lithuania**	**PX642812**
Didymocyrtis pini	CAA 1002		Portugal	MW732246
Didymocyrtis pini	**73 G-280**	***Pacifastacus leniusculus*, liver**	**Lithuania**	**PX622612**
Epicoccum tritici	**MFLUCC 16-0598**		**Kingdom of Thailand**	**KX965723**
Epicoccum tritici	**39 G-287**	***Astacus astacus*, muscle**	**Lithuania**	**PX642685**
Fusarium avenaceum	HGUP190194		China	MZ724839
Fusarium avenaceum	**42 G-249**	***Astacus astacus*, muscle**	**Lithuania**	**PX642692**
Fusarium equiseti	CBS 307.94		The Netherlands	MH862468
Fusarium equiseti	**27 G-252**	***Faxonius limosus*, muscle**	**Lithuania**	**PX633167**
Fusarium foetens	CBS 110286		The Netherlands	NR_159865
Fusarium foetens	**4 G-253**	***Astacus astacus*, liver**	**Lithuania**	**PX642815**
Fusarium foetens	**3 G-254**	***Astacus astacus*, muscle**	**Lithuania**	**PX642691**
Fusarium foetens	**1 G-255**	***Astacus astacus*, muscle**	**Lithuania**	**PX642690**
***Fusarium* sp.**	**45 G-248**	***Astacus astacus*, liver**	**Lithuania**	**PX642816**
Fusarium sporotrichioides	102E		Poland	MT732817
Fusarium sporotrichioides	**70 G-250**	***Pacifastacus leniusculus*, muscle**	**Lithuania**	**PX622895**
Fusarium sporotrichioides	**72 G-251**	***Pacifastacus leniusculus*, liver**	**Lithuania**	**PX622611**
Microdochium bolleyi	KAS1350		The Netherlands	KP859018
Microdochium bolleyi	**74 G-279**	***Pacifastacus leniusculus*, liver**	**Lithuania**	**PX622613**
Neocucurbitaria cava	CBS 257.68		The Netherlands	MH870841

Neocucurbitaria cava	**30 G-272**	***Pontastacus leptodactylus*, liver**	**Lithuania**	**PX640730**
***Neocucurbitaria* sp.**	**26 G-273**	***Faxonius limosus*, muscle**	**Lithuania**	**PX633163**
***Neocucurbitaria* sp.**	**15 G-274**	***Faxonius limosus*, muscle**	**Lithuania**	**PX633162**
Neodidymelliopsis negundinis	MFLU:16-1123		Italy	MT185531
***Neodidymelliopsis* sp.**	**55 G-285**	***Faxonius limosus*, muscle**	**Lithuania**	**PX633159**
Neosetophoma sp.	FeC133		Poland	MW446977
***Neosetophoma* sp.**	**61 G-283**	***Faxonius limosus*, liver**	**Lithuania**	**PX632641**
Ophiognomonia intermedia	2048		United Kingdom	DQ323540
Ophiognomonia intermedia	**16 G-277**	***Faxonius limosus*, muscle**	**Lithuania**	**PX633161**
Paracamarosporium fagi	CPC 24892		The Netherlands	KR611887
Paracamarosporium fagi	**63 G-282**	***Pacifastacus leniusculus*, muscle**	**Lithuania**	**PX622894**
Paraphaeosphaeria neglecta	CBS 434.71A		The Netherlands	JX496090
Paraphaeosphaeria neglecta	**82 G-278**	***Pontastacus leptodactylus*, liver**	**Lithuania**	**PX640729**
Paramyrothecium humicola	CBS 127295		The Netherlands	MH864508
***Paramyrothecium* sp.**	**54 G-291**	***Astacus astacus*, liver**	**Lithuania**	**PX642809**
Penicillium bialowiezense	CBS 227.28		The Netherlands	NR_165994
***Penicillium* sp.**	**80 G-256**	***Pontastacus leptodactylus*, muscle**	**Lithuania**	**PX640731**
***Penicillium* sp.**	**58 G-257**	***Faxonius limosus*, liver**	**Lithuania**	**PX632643**

***Penicillium* sp.**	**50 G-258**	***Astacus astacus*, liver**	**Lithuania**	**PX642814**
***Penicillium* sp.**	**43 G-259**	***Astacus astacus*, muscle**	**Lithuania**	**PX642689**
***Penicillium* sp.**	**24 G-260**	***Faxonius limosus*, muscle**	**Lithuania**	**PX633166**
***Penicillium* sp.**	**18 G-261**	***Faxonius limosus*, muscle**	**Lithuania**	**PX633165**
***Penicillium* sp.**	**17 G-262**	***Faxonius limosus*, liver**	**Lithuania**	**PX632642**
Phoma herbarum	CBS 615.75		The Netherlands	KF251212
Phoma herbarum	**8 G-268**	***Astacus astacus*, liver**	**Lithuania**	**PX642811**
Phoma macrostoma	KRP10-2		Lithuania	HM036611
***Phoma* sp.**	**53 G-265**	***Astacus astacus*, muscle**	**Lithuania**	**PX642687**
Septoriella pseudophragmitis	CBS 145417		The Netherlands	MK560161
Septoriella pseudophragmitis	**52 G-286**	***Astacus astacus*, liver**	**Lithuania**	**PX642810**
Tolypocladium pennsylvanicum	CBS 152275		The Netherlands	NR_199123
***Tolypocladium* sp.**	**69 G-281**	***Pacifastacus leniusculus*, liver**	**Lithuania**	**PX622609**
***Tolypocladium* sp.**	**G-290**	***Pacifastacus leniusculus*, liver**	**Lithuania**	**PX622615**
Trichoderma sp.	KUMCC 21-0805		China	ON426894
***Trichoderma* sp.**	**56 G-284**	***Faxonius limosus*, muscle**	**Lithuania**	**PX633160**
Outgroup				
Sacharomyces cerevisiae	CBS 1171		USA	NR_111007
